# Glances at the Hospitals

**Published:** 1904-10-01

**Authors:** 


### GLANCES AT THE HOSPITALS.

### GLASGOW ROYAL INFIRMARY.

The ordinary revenue was £29,063, and the ordinary expenditure was £37,093, and in addition £1,276 was handed over to the funds of the convalescent home. The extraordinary revenue was £22,436, [and this was dealt with as follows:—To extraordinary expenditure £3,416, to the ordinary expenditure £9,306, and to stock account £9,713. The financial statement on page 13 shows that the annual subscriptions amounted to £10,016, employees in public works contributed £8,353, Hospital Sunday produced

£2,524, and students' fees £357. It is proposed to reconstruct the infirmary, and "an important step will very soon be taken, at the instance of the Lord Provost, for obtaining the necessary funds to enable the managers thereafter to proceed with the entire undertaking." From the medical tables we find that 64 cases of acute rheumatism were treated, and that 52 of these were cured, 9 were relieved, 1 was discharged not improved, and 2 died. Of pneumonia 168 cases were treated, showing the following results: 107 cured, 8 relieved, 8 not relieved, and 44 died. Of 11 primary amputations of arm, 10 recovered and 1 died. Of 10 amputations of the thigh, all recovered. Of 10 cases of lithotomy, 9 recovered and 1 died. Of 18 cases of ovariotomy, 17 recovered and 1 died. The infirmary contained 528 patients on the first of January, 7,271 were admitted during the year, 527 remained on the last day of December, and 7,272 were treated to a termination. The average number resident was 544, the average stay in the infirmary was 27 days, and the death-rate was 12·1 per cent. among the men, and 9·8 among the women. There is no anæsthetic table.

### SOUTH DEVON HOSPITAL, PLYMOUTH.

The ordinary income amounted to £7,532 and the ordinary expenditure to £9,038, but of this sum upwards of £700 was spent on repairs and on keeping up the grounds. Nevertheless there was a deficit of £1,506 and the committee say "they cannot ignore the grave considerations involved in this fact and in the possibility of its recurrence in future years." The hospital has an assured income of £2,700 a year, derived from funds invested, and its annual subscriptions amounted to £1,633. We notice from the table on page 25 that the contributions of patients amounted to £193 and that the private nursing institution produced £191, but we do not notice how much the workpeople themselves contributed to the funds of the hospital. The average number of beds occupied rose from 104 in 1902 to 115 in 1903, and the average cost per bed occupied was £78 11s. The average stay in the hospital was 31 days, and the average cost per patient was £6 15s. 3d. At the beginning of the year 105 patients remained in the hospital and 1,231 were admitted during it, making a total of 1,336 under treatment. Of these 854 were discharged cured, 87 greatly relieved, 156 relieved, 55 not relieved, 90 remained in the wards, and 94 died, but the latter number contained many cases taken to the hospital in a dying state. The table of operations is divided into columns showing the "totals", the "cured," the "relieved," and the "died." There were 21 cases of ovariotomy, of whom 18 were cured, 1 relieved, and 2 died. The operation for the radical cure of hernia was performed 48 times, all being successful; 755 operations were performed on in-patients and 595 on the out-patients, but no anæsthetic table is given. The medical tables merely show the numbers treated for the various diseases, and, therefore, might just as well have been omitted.

### THE LEEDS HOSPITAL FOR WOMEN AND CHILDREN.

The ordinary income was £1,294, and donations amounted to £790. The expenditure was £2,209. The admissions of the year were 356 women and 31 children. The average number of beds occupied was 25, and the average stay in the hospital was 22 days. The percentage of deaths was 3 20. The table of operations shows the disease, the nature of the operation, and the result. Altogether there was a total of 415 operations performed during the year, and of these 406 were successful, and in 9 death followed. The anæsthetic table shows that ether was the agent used in 315 instances, chloroform in 77, chloroform and ether in 7, ethyl chloride and ether in 13, ethyl chloride in 2, and cocaine in 1.

### MACCLESFIELD GENERAL INFIRMARY.

The income for the year amounted to £2,967, and the expenditure to £3,782, showing a deficit on the year's working of £815; and the Board say this is largely accounted for by the increased expenditure in drugs and surgical instruments rendered necessary by the increasing number of serious cases and by the increased cost of necessaries. It is further pointed out that the Infirmary is now thirty years old, and that many renewals have been unavoidable. On the other hand, we notice that the endowment fund has been increased during the year by £7,000, and another £1,000 has been left to endow a bed. Still, with £1,442 due to the bank it will be necessary to reduce expenses or find fresh subscribers. At the beginning of the year there were 40 patients in residence, and 478 were admitted during the year, making a total of 518 under treatment. An analysis of medical and surgical cases is given which shows the numbers treated and the numbers dying of the various diseases; but the operation table merely gives the numbers operated on for the particular diseases and shows no results. As there are two house-surgeons and a daily average of patients which probably does not exceed 50, we think the tables might have conveyed fuller information. The same may be said of the secretary's part of the report. We could not find any statement of the cost per bed occupied or of the cost per head per week.

### VICTORIA HOSPITAL, BLACKPOOL.

The income amounted to £1,862, and the expenditure to £2,262, showing a deficit of £400; but nearly half of that sum was standing over from the previous year. The Board remark that the increased work of the hospital entails larger expenditure, and they regret that this is not met by increased income. The annual subscriptions amount to £566, but it does not appear from the summary on page 50 how much of this is subscribed by the workpeople themselves. The collecting-boxes produced £218 towards the income, and no doubt much of this sum was given by the workpeople. At the beginning of the year the wards contained 36 patients, and 438 were admitted during the year, which shows an increase of 11 per cent. on the previous year. The average stay in the hospital was 28 days. This can hardly be described as short, but we are glad to be able to state that it is three days shorter than in the previous year. The average daily number of beds occupied was 36, and the average death rate was 8·5 per cent. The surgical operations were 177 in number, as against 116 of the previous year. The medical and surgical tables are very well prepared, and give columns for the "recovered," "relieved," "died," "remaining," and for "remarks." Of 14 cases of pneumonia 12 recovered and 2 died. Oddly enough, the operation table does not give results. We note that chloroform was the anæsthetic used in 166 of the operations and ether in 11.

### NEWTON ABBOT HOSPITAL.

At this little hospital the income amounted to £1,597, and the expenditure to £1,446, showing a balance in hand of £151, and giving a lesson in economy and management to many larger institutions. There were 262 patients admitted to the wards during the year, as against 221 in the previous year. Among the number were 17 private patients, being 10 more than in the year before. There were 8 deaths, but 6 were in a dying state when admitted. A complete register of patients is printed, and gives all desirable information of every patient under treatment individually, a plan which is admirable for tracing any particular patient, but which does not admit of such ready comparison with other hospitals as a carefully prepared table would.

